# From machine learning to digital twin integration for livestock production and research

**DOI:** 10.3389/fvets.2026.1744053

**Published:** 2026-02-02

**Authors:** Mohamed Abdelrahman, Sali Issa, Montaser Elsayed Ali, Jamal Alotaibi, Fahad Alshanbari

**Affiliations:** 1 Animal Production Department, Faculty of Agriculture, Assuit University, Asyut, Egypt; 2 Department of Electrical Information of Science and Technology, Hubei University of Education, Wuhan, China; 3 Department of Animal Production, Faculty of Agriculture, Al-Azhar University, Assiut, Egypt; 4 Department of Computer Engineering, College of Computer, Qassim University, Buraydah, Saudi Arabia; 5 Department of Medical Biosciences, College of Veterinary Medicine, Qassim University, Buraydah, Saudi Arabia

**Keywords:** animal behavior analysis, digital twin, health, livestock production, care, Machine Learning, predictive analytics

## Abstract

Globally, climate change, economic crises, and increased food demand pose significant challenges to the stability of agricultural production systems, underscoring the urgent need for more innovative approaches and tools to advance livestock production science. Machine Learning (ML) development supported the Digital Twin (DT), a digital replica of a real-world entity, as a game-changer in modern livestock science, enabling the prediction, optimisation, and simulation across various research environments. At the same time, it has been shown that synergism between ML and Digital Twin (DT) can mimic animals' physiological and physical state and behavior based on input data, leading to a better understanding of animal behavior, nutritional requirements, physiological status, or environmental stressors to investigate responses and suggest precise decisions. Moreover, such animal simulation models can offer deeper insights and predictive analytical tools that support animal welfare, forecast production efficiency, and sustainability. Although traditional simulation models are mainly snapshot-state models that indicate what should happen on average, ML-DT integration serves as a living mirror, dynamically predicting what is happening right now and what will happen to each animal under various changes. This integration can be a versatile tool for introducing solutions in the research domain; however, its augmentation remains complex and poses significant ethical, economic, and governance challenges. This review discusses recent ML-DT synergism applications in both barns and labs, highlighting their potential to reform both industry and research.

## 1 Introduction

Recently, Machine learning (ML) inclusion in animal science has grown significantly, supported by the development of remote monitoring technologies while facilitating more sustainable resource utilization by encompassing a range of tools and techniques, including in-field livestock monitoring, greenhouse gas emissions, body composition and physiology assessments, ground- or aerial-based livestock, automated in-field live weight measurement, on- and in-animal devices, and GPS (1, 2).

Additionally, ML models like random forest (RF), neural network (NN), deep learning (DL), Ensemble, support vector machine (SVM), k-nearest neighbors (KNN), and logistic regression (LR) showed promising results, predict outcomes, and uncover patterns crucial for enhancing animal health, productivity, and welfare (3–5). Also, these models can forecast economic returns without requiring extensive long-term individual observations (6), milk prices (7), and beef and lamb prices, offering robust support for managing these prices and facilitating higher income for producers (8).

**Figure 1:**
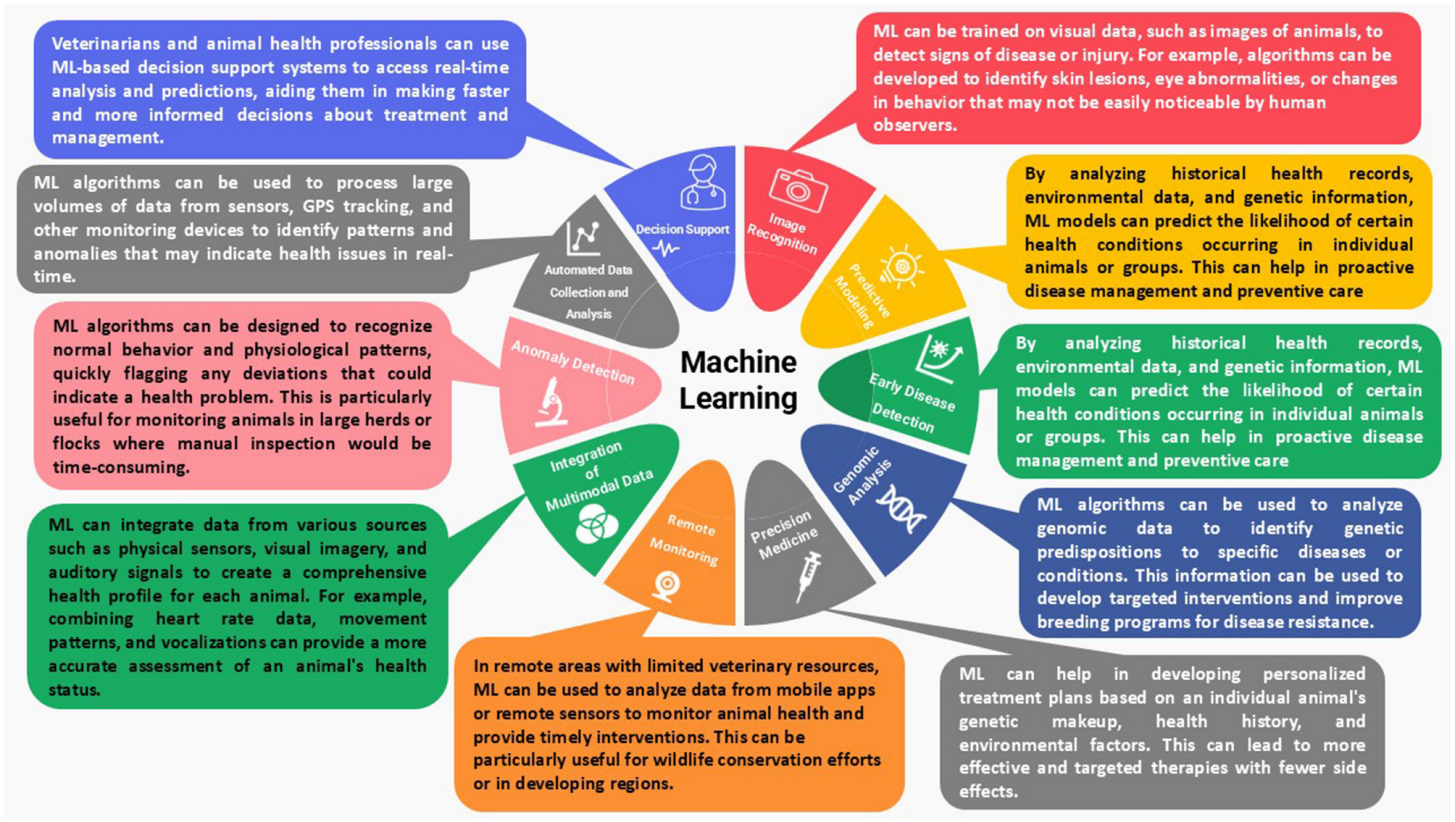
Machine-learning applications in livestock production and research.

These ML applications demonstrate the versatility in addressing complex problems not only in the production sector, but also in research fields such as artificial neural networks (ANN), which have been deployed in various biological sciences fields for data categorization into different classes, pattern recognition, future prediction, performance optimisation, and decision-making support (9) (Figure 1). Then, the next generation of ML models merged to introduce the digital twin (DT), which virtually constructs a digital replica that reflects the characteristics, state, and behavior of the corresponding physical entity and is updated in real-time as the physical entity changes (10–12). Furthermore, ML–DT integration has been applied to multiple domains, including nutrition, health, behavior, and product quality, such as predicting *in vitro* rumen VFA production (13), developing digital twin models for cattle care (14), and evaluating meat quality using computer vision (15). Additionally, such advancements led to the Precision Livestock Farming (PLF) approach, which incorporates Information and Communication Technologies (ICT) to enhance agricultural practices, reduce costs, and increase production, significantly contributing to the industrial revolution, referred to as Industry 4.0 and 5.0 (16–18).

Therefore, progressive advances in integrating ML-DT have opened up the scope for digital tools to solve industrial and scientific challenges that may transform livestock production and research, both in the present and the future.

## 2 Review methodology

### 2.1 Review design and scope

This review employs a narrative review methodology to critically synthesize recent research on machine learning (ML) and digital twin (DT) technologies and their augmentation in livestock production and research. With a focus on precision livestock farming, decision-support systems, and digital transformation, the scope includes methodological advancements, application domains, and integration issues across poultry, ruminant, pig, and multi-species livestock systems. The review investigates the applications and the potential for extending this contribution to the livestock research field.

### 2.2 Literature sources, search strategy, and selection process

The references were extracted from peer-reviewed articles indexed in major international scientific databases, including Google Scholar, Web of Science (SCI-Expanded), Scopus, ScienceDirect, SpringerLink, IEEE Xplore, PubMed, PMC, MDPI journals, and Frontiers journals.

“The review mainly focused on original research and review articles, complemented by selected studies for advanced methodological. While searches were in English, the keywords used for relevant articles, including but not limited to: “machine learning,” “deep learning,” “artificial intelligence,” “digital twin,” “precision livestock farming,” “smart farming,” “sensor- based monitoring,” “IoT,” “genomic prediction,” “animal health monitoring,” and “livestock decision support systems.” The initial selection focused on titles and abstracts to eliminate irrelevant records and duplicates. The final dataset comprised 195 references, representing a comprehensive, multi-species, and multidisciplinary set of articles (Tables 1, 2).”

**Table 1:** The 195 references distribution by journal/source.

Journal/Source	No. of articles
Animals (MDPI)	34
Sensors (MDPI)	17
Agriculture/Electronics/Applied Sciences (MDPI)	14
Journal of Animal Science	12
Computers and Electronics in Agriculture	11
Journal of Dairy Science	9
Poultry Science	9
Frontiers Journals (Vet Sci, Anim Sci, AI, Genomics)	14
Scientific Reports	8
Animal/Livestock Science/Meat Science	14
Genetics/Genomics Journals	11
AI/Data Science/Engineering Journals	11
Conference Proceedings (IEEE, IFAC, others)	7
Books/Book Chapters (Springer, Elsevier)	4
Other journals (single-occurrence sources)	20
The total	195

**Table 2:** Overall summary by livestock category.

Category	Number of references
Poultry	48
Ruminants	79
Pig/multi-species	68
Total	195

## 3 Mathematical model to machine learning: theory-driven vs. data-driven competition or integration?

Mathematical models (MMs) in livestock production have been utilized to simulate and forecast various aspects of behavior, health conditions, reproductive status, production performance, and environmental factors (19–21). Although MMs are difficult to reproduce, which exacerbates the challenge of automatically updating key input parameters (22, 23), MMs are still more transparent, Easier to audit, and safer from algorithmic bias than ML, which is expanding and competing with traditional mathematical restrictions (24–26). At the same time, working with larger datasets posed the main challenge for MMs' application, as addressing accuracy issues requires simplifying assumptions (27–29). However, ML models showed remarkable accuracy and potential in predicting production outcomes and health alerting models (30) (Table 3).

**Table 3:** Conceptual, strengths, limitations, comparison of machine learning (ML), and mathematical models (MM) used in livestock research.

**Comparison**	**ML**	**MM**	**References**
Approach	Artificial intelligence subsets enable systems to involve training algorithms, identify patterns, and make decisions with minimal human intervention, using large datasets to improve their performance over time.	They are used to understand the relationships between variables and predict outcomes based on established mathematical principles and theory.	(75, 76)
Data processing	ML algorithms require substantial data processing capabilities, particularly those for deep learning. The need for large volumes of data and the iterative nature of training often require significant computational resources.	These models process data using predefined equations and relationships, which are less data-dependent and require less processing power. The data requirements are often specific and may not need to be as large as those for ML models.	(76)
Complexity and adaptability	By retraining, ML algorithms can handle complex, high-dimensional data and adapt to new data, which may be less computationally intensive than redesigning a mathematical model.	While complex, they often require a deep understanding of the underlying processes to be accurately formulated.	(76)
Development process	The development process involves data preprocessing, feature selection, model selection, training, and validation.	The process involves defining the problem, formulating equations based on theoretical knowledge, solving these equations, and validating the model against known data.	(77)
Real-time processing	Real-time ML processing can be more challenging, especially with large models, as it may require substantial computational power to process data and make predictions promptly.	Since mathematical models are often based on simple calculations, they can be processed in real time, making them suitable for applications requiring immediate responses.	(78)
**Limitations**			
	•ML models, especially deep learning models, can be considered “black boxes” because it's often difficult to understand how they arrive at their predictions. Compared to ML, MM models are generally more transparent and interpretable because their structure and variable relationships are explicitly defined.	•Scalability can be an issue if the model's complexity grows with the problem's size, leading to computational challenges.	(78–80)
	•MLs are heavily dependent on data. They require large amounts of data to train and improve their accuracy.	•Adapting them to new data or conditions may require a complete reevaluation and reformulation.	
	Wearable sensors, imaging systems, and automated monitoring platforms produce high-resolution data streams that require substantial computing resources, including reliable internet access and high-performance hardware. For livestock systems in low- and middle-income areas, where technological infrastructure and qualified workers may be limited, these criteria pose serious challenges. The ongoing maintenance and updates of ML models also drive long-term operational costs.	•While data can be used to parameterise these models, MM are not solely reliant on data. They are based on theoretical understanding and can be developed with or without empirical data.	

## 4 Data source and model initiation; the record-based, sensor-based, and the challenging integration

**Figure 2:**
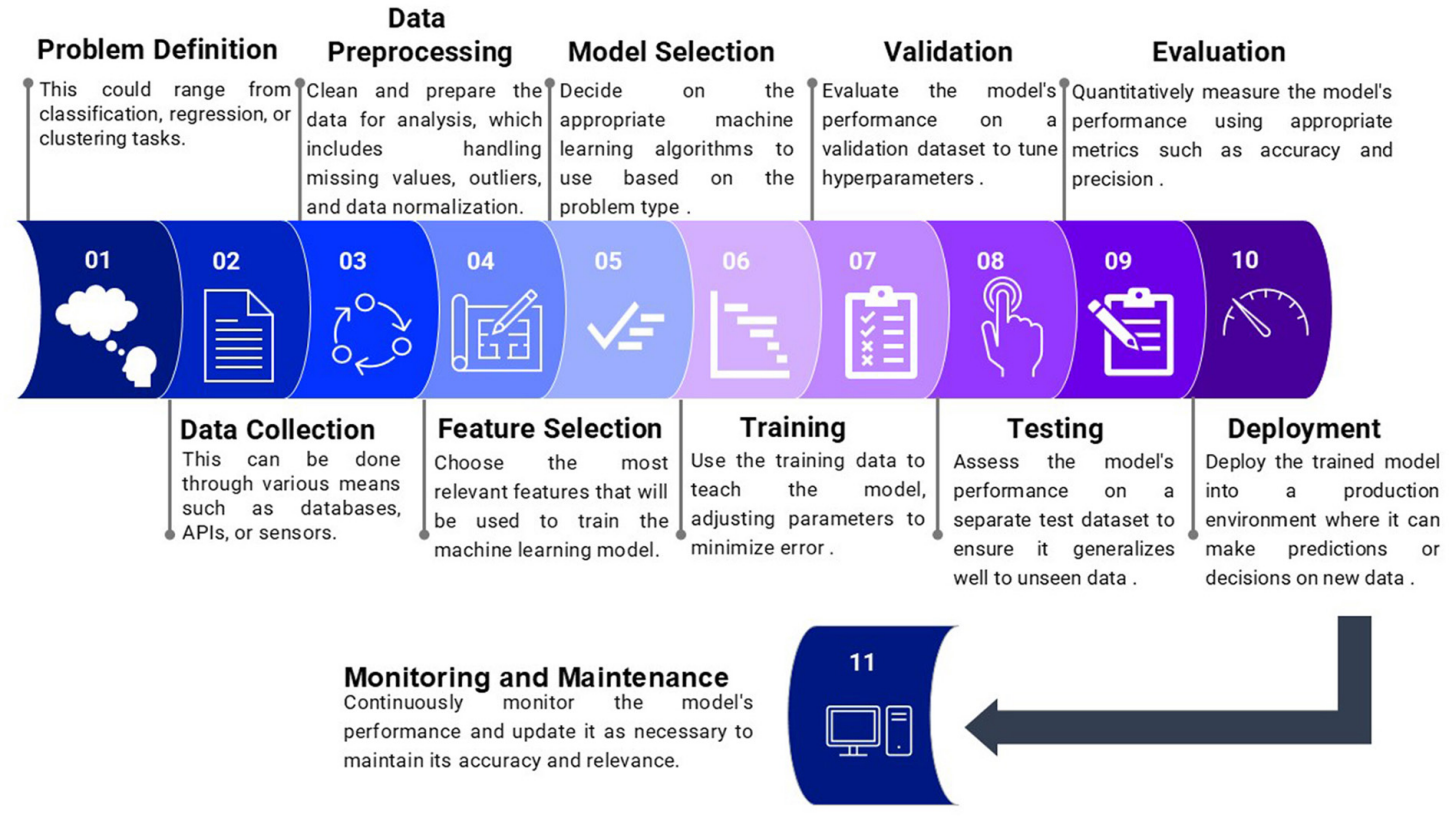
Describes initiating a machine learning model.

Before adopting a specific model, problem identification with parametric, scalable attributes to be evaluated is the critical first step for model building; then, the process proceeds to subsequent steps (Figure 2). Then ML processes large datasets, identifies intelligent patterns, and makes predictions based on learned experience (31, 32), depending on farm records such as milk yield, milk analysis, feed records, reproduction records, breeding records, and health records (33, 34).

However, researchers should incorporate extensive variability into their datasets and employ classifiers to mitigate overfitting, which can be controlled through cross-validation (31, 32) and regularization (35, 36), both of which are applied in livestock production and prediction studies (37, 38).

To track behavior, health, and production in real time, smart collars for dairy cows incorporate a range of wearable technologies, including accelerometers, GPS, RFID, and microphones (39). IoT-enabled neck collars with activity and temperature sensors lessen reliance on labor and enable early identification of health abnormalities (40). Furthermore, collar systems that integrate wireless communication, GPS, and vital-sign detection demonstrate how real-time livestock tracking and health monitoring can be implemented in practice (41).

Herein, the sensors serve as a significant data source for innovative farming models (42), including behavioral (43–47), physiological (48–52), and environmental (53–56), which raises integration challenges between record-based and sensor-based data (57).

**Figure 3:**
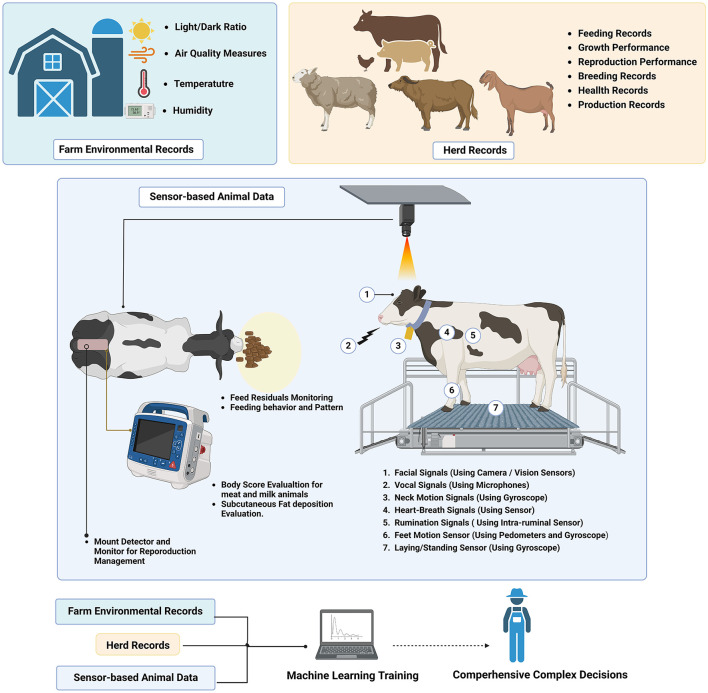
Integrating different sensor-based and record-based data in the ML model.

Therefore, to tackle these challenges, some reports indicate that processes must be objective-driven and specifically tailored to the intended behaviors for specific farm applications (58) (Figure 3). So this challenge highlights the importance of model selection, in which analytical frameworks and algorithms must align with the targeted objective and the farm environment (59) (Table 4).

**Table 4:** Application of different ML models through different data and species.

**Data type**	**ML model**	**Modeling output**	**Species**		
			**Ruminants**	**Pigs**	**Poultry**
Production records, feed intake, phenotypic traits.	regularizing models	Production and genomic performance prediction	(44–48, 81, 82)	(49–55)	(56, 83–87)
Sensor data, farm records, health, and management data	Tree-based models	Condition estimation and behavior classification, risk monitoring, and assessment	(88–91)	(92–97)	(98–102)
Accelerometer data, sample spectra, and imaging features.	Support Vector Machines (SVM)	Animal status, carcass, and body condition scoring	(103–108)	(49, 53, 55, 109–112)	(100, 113–116)
Production, metabolic, and environmental data.	Artificial Neural Networks (ANN)	Production and reproduction traits, and environmental impacts, Prediction	(117–126)	(54, 127–135)	(102, 136–140)
Visual/time-series and sensor data.	Deep learning models	Visual identification, status monitoring, and early detection.	(141–149)	(150–158)	(116, 159–165)
Multi-sensor data, omics datasets.	Unsupervised learning	Animal phenotyping, anomaly detection,	(166–171)	(154, 172–177)	(178–184)

## 5 Machine learning-digital twin augmentation in livestock production and research

The relationship between ML and DT is jointly integrated, with ML, especially deep learning, providing digital twins with robust data analysis and pattern recognition capabilities, enabling more intelligent and adaptive applications (60–62). At the same time, DT can enhance predictive analytics, automate decision-making, and ensure secure data exchange among stakeholders when combined with cutting-edge AI/ML, blockchain, and reinforcement learning (12, 62).

For instance, ML-DT integration in comparative genomics can be advantageous for pinpointing virtual phenotypes of livestock traits important for genetic selection and responses under different conditions, thereby helping identify phenotypes linked to resilience and productivity (63, 64). This integration facilitates more effective breeding, nutrition, and sustainability studies by supporting enhanced phenotyping, forecasting production outcomes, and investigating host-environment interactions (62, 65, 66). Additionally, DTs offer a platform for *in silico* experimentation, allowing researchers to evaluate scenarios about resource utilization, disease transmission, and climate adaptation with lower ethical and financial risk (67, 68).

- **Support real-time monitoring and feedback**: Digital twins can use machine learning algorithms to monitor and adjust the simulation of physical entities in real time (11, 69).
- **Predicting scalability and flexibility**: Digital twins can be integrated with machine learning operations (MLOps) platforms in complex production environments to enable more intelligent and automated decision-making. DT can commence with basic configurations that can progressively be augmented with additional ML models to strengthen their intelligence and autonomy (60, 62).
- **Reduce costs and improve efficiency**: Efficient Digital twins combined with ML algorithms can reduce research costs and improve resource utilization efficiency. This combination can achieve higher responsiveness, predictability, and adaptability by managing the full life cycles of different livestock species, biological processes, and farm practices. Then it can depict a research scene more richly, with more possible interactions and more extended indirect relations that can support a deeper, more precise evaluation of research outputs (70).
- **Cross-domain applications**: The combination of digital twins and ML is not limited to farm production applications; it can also be applied across multiple fields. Simulation constitutes information fusion, as it amalgamates and enhances data from several heterogeneous sources. DT analyses physical twins from diverse viewpoints, using various data sources and evaluating the potential consequences of actions. Information fusion and the ongoing nature of operations illustrate the comprehensive status of past and present system conditions, facilitating the projection of future states (71).
- **Environmental control**: Digital twins can simulate various research conditions and environments, and machine learning can adjust control parameters in the actual environment based on these simulations to improve animal comfort and production efficiency (72).

Also, there is promising potential for DT application in research by integrating physical research with a robust virtual model to extend research results and broaden the scope of physical research; while ML provides digital twins with intelligent analysis and prediction capabilities, digital twins afford ML with a highly detailed, up-to-date data environment. This DT fusion will bring more innovation and change to livestock research in the future (Table 5).

**Table 5:** Examples of ML studies and the potential of digital twin to develop the results outcomes.

**Animal species**	**Study**	**Studied variable**	**Marker**	**References**	**DT upgrading potential**
Dairy cattle	Developing an artificial neural network for the early prediction of subclinical ketosis during lactation.	Subclinical Ketosis	50,025 and 10,005 SNPs	(185)	Predict the effect on lactation performance and yield, and forecast culled animals.
Beef cattle (Nellore)	Utilizing machine learning to find small subsets of biologically significant genes for classifying animals into High Feed Efficiency and Low Feed Efficiency categories.	Feed efficiency	16,423 genes	(186)	Rearranging this subset or investigating new gene subsets to predict their effect on the traits.
Goat	Efficiently oversee the health and welfare of their goats, thereby enhancing living circumstances and augmenting dairy output.	Animal behavior	Goat activity	(187)	Mimic population behavior changes and track their effect on herd performance.
Dairy cattle	Image processing algorithms and the YOLOv8 model facilitate the real-time, non-invasive monitoring of feeding periods.	Feed utilization efficiency	Feeding pattern	(188)	Simulate different feeding strategies and forecast productivity and economics.
Cattle and Buffalo	Forecasting lumpy skin disease infection	LSD occurrence	Meteorological and geological attributes	(189)	Stimulate assessment of infection virulence and its effects on productivity, and estimate potential economic losses in specific regions.
(Sheep) (Harnai)	Predicting live weight at the post-weaning period.	Growth performance	Body biometric parameters and sex factor.	(190)	Predicting the Economics of the Production
Pigs	Infection prediction in swine populations, both seven and 30 days in advance	Infection Outbreak	Nearby farm density, historical test rates, piglet inventory, feed consumption during gestation, and wind speed and direction.	(95)	Simulate the evaluation for disease prevention and mitigation strategies.
Cattle	16S rRNA sequencing and machine learning methodologies identified a dozen species as taxonomic indicators for distinguishing infection.	The *Mycobacterium avium* disease state.	Fecal microbiota	(191)	Investigate the effects of microbial dysbiosis on pathogenic microbes. The Gut-host interaction
Chicken	Investigate antimicrobial resistance profiles across multiple chicken farms and abattoirs.	Antimicrobial resistance genes	*E. coli*	(192)	Forecast the intensity of ARGs under different environmental conditions and correlate ARGs with different microbiomes.
*In-vitro* fermentation	Prediction of Methane Production from *in vitro* Ruminal Fermentation	Methane	Volatile fatty acids	(193)	Introducing a cross-species Virtual rumen fermentation model
Water-Deer Water Buffalo Sheep Buffalo	Combined network analysis and interpretable machine learning reveal	Environmental adaptability	Microbial genomes	(194)	Predicting which pathogen species are most likely to emerge in the future
Wild-Livestock	ML demonstrates one approach to planning for and preventing disease emergence in livestock.	Pathogen-host associations at the wildlife–livestock interface	Bacterial association	(195)	

Herein, a meaningful question will be posed: How can ML-DT transform livestock science, and what can be introduced into the field of research?

**Figure 4:**
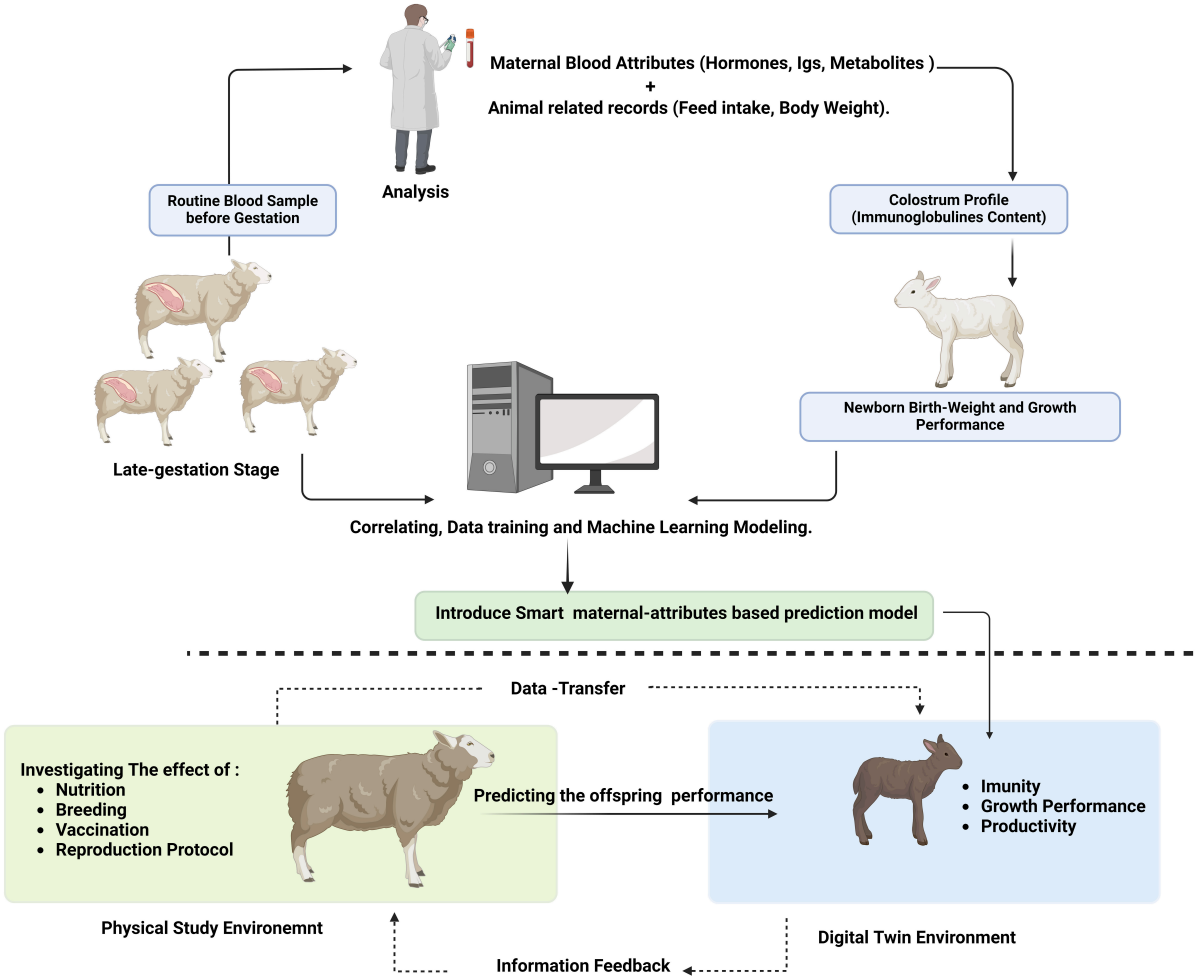
Describes the virtual lab model in extending the research findings.

For example, if a physical experiment investigating the maternal nutrition effect on reproductive physiology and hormonal regulation is piloted at this stage, the results will investigate a single research question. But what if we want to explore the further effects on offspring growth virtually? Can we deploy an ML-DT module to predict colostrum quality and offspring immune response using prior data linking hormonal effects to colostrum quality and offspring performance? Figure 4. The next stage can be developed by integrating multiple DT models to predict birth weight and, subsequently, mature body weight, average daily gain, and feed efficiency. In this hybrid research environment (physical-virtual), researchers can extend physical research findings using a virtual assistant, which we can call the virtual lab. Although model fitness for the virtual part will be challenging and critical, the model can produce more results with fewer physical resources, less time, and fewer specialized research environments. Moreover, the central role of this concept, “co-valorisation,” is to connect previous scientific work and data records with new golden opportunities to launch a contemporary era with powerful outputs and findings well positioned to reshape livestock production and research.

- **Challenges faced by ML-DT in augmentation and applications**. To fully expand ML-DT integration through Smart Livestock Farming (SLF) across livestock species, a policy that encourages substantial equipment investment is critical. Also, data produced by SLF can be sensitive, necessitating robust legal measures to ensure information security and enhance trust in data sharing. Establishing an SLF big data recirculation center is essential, as individual farms often lack the capacity to process large volumes of data. Additionally, integrating public and farm data with AI and ML can leverage SLF big data to create digital twins, potentially increasing the economic value of the livestock industry through advanced simulations (73).
  In addition to data accuracy, data synchronization, real-time analytics, computational load, and calibration issues, model suitability is a critical factor in building confidence in these models. Regarding farm-applicability constraints such as cost, scalability, hardware requirements, farm size, and on-farm feasibility, they clash with farmer acceptance and uncertainties, which may hinder wider deployment. Therefore, the transition from ML models to partially ML-DT-integrated and fully integrated models, adaptable across different research and farm environments, may be a key gateway to transforming livestock production and research (74).
  However, despite the previous challenges and the limited number of proposed integrated models, most of which are at the conceptual stage, the gradual adoption and transition from physical to virtual, coupled with a multi-model approach, may be highly promising for avoiding future obstacles.

## 6 Conclusion

Integrating ML with DT into livestock production research, from predicting growth performance to understanding the relationships between health, behaviors, and different physiological statuses, to biological simulation models, can uncover hidden insights. Although ML-DT integration can completely transform the nature of outputs from production and research data, the integration models remain conceptual and limited due to data governance and resource utilization measures. There is an urgent need for more applications across both research and production plateaus, considering the variation in species, data types, and sources, and model selection and suitability. Such cross–domain models can reduce the time and resources required for more in-depth livestock research and production, enabling seamless transitions between physical/virtual and virtual/virtual environments. Although the early findings are promising and open the door to co-valorizing research data, these applications need to be comprehensively designed and integrated to solve sustainable livestock science and practices challenges.
