# Correction: From machine learning to digital twin integration for livestock production and research

**DOI:** 10.3389/fvets.2026.1810873

**Published:** 2026-03-31

**Authors:** Mohamed Abdelrahman, Sali Issa, Montaser Elsayed Ali, Jamal Alotaibi, Fahad Alshanbari

**Affiliations:** 1 Animal Production Department, Faculty of Agriculture, Assuit University, Asyut, Egypt; 2 Department of Electrical Information of Science and Technology, Hubei University of Education, Wuhan, China; 3 Department of Animal Production, Faculty of Agriculture, Al-Azhar University, Assiut, Egypt; 4 Department of Computer Engineering, College of Computer, Qassim University, Buraydah, Saudi Arabia; 5 Department of Medical Biosciences, College of Veterinary Medicine, Qassim University, Buraydah, Saudi Arabia

**Keywords:** animal behavior analysis, digital twin, health, livestock production, care, Machine Learning, predictive analytics

There was a mistake in Tables 1 and 2 as published.

In Table 1, one category was missing. In Table 2, the total reference number was updated. The corrected tables appears below.

**Table 1:** The 195 references distribution by journal/source.

Journal/Source	No. of articles
Animals (MDPI)	34
Sensors (MDPI)	17
Agriculture/Electronics/Applied Sciences (MDPI)	14
Journal of Animal Science	12
Computers and Electronics in Agriculture	11
Journal of Dairy Science	9
Poultry Science	9
Frontiers Journals (Vet Sci, Anim Sci, AI, Genomics)	14
Scientific Reports	8
Animal/Livestock Science/Meat Science	14
Genetics/Genomics Journals	11
AI/Data Science/Engineering Journals	11
Conference Proceedings (IEEE, IFAC, others)	7
Books/Book Chapters (Springer, Elsevier)	4
Other journals (single-occurrence sources)	20
The total	195

**Table 2:** Overall summary by livestock category.

Category	Number of references
Poultry	48
Ruminants	79
Pig/multi-species	68
Total	195

There was a mistake in the caption of Table 1 as published.

The corrected caption of Table 1 appears below.

“Table 1. The 195 references distribution by journal/source.”

In Section 2.2, Paragraph 2, there was a mistake in the total reference number.

This section has been corrected:

“The review mainly focused on original research and review articles, complemented by selected studies for advanced methodological. While searches were in English, the keywords used for relevant articles, including but not limited to: “machine learning,” “deep learning,” “artificial intelligence,” “digital twin,” “precision livestock farming,” “smart farming,” “sensor- based monitoring,” “IoT,” “genomic prediction,” “animal health monitoring,” and “livestock decision support systems.” The initial selection focused on titles and abstracts to eliminate irrelevant records and duplicates. The final dataset comprised 195 references, representing a comprehensive, multi-species, and multidisciplinary set of articles (Tables 1, 2).”

The original version of this article has been updated.

